# Angiogenesis in a 3D model containing adipose tissue stem cells and endothelial cells is mediated by canonical Wnt signaling

**DOI:** 10.1038/boneres.2017.48

**Published:** 2017-12-13

**Authors:** Xiaoxiao Cai, Jing Xie, Yang Yao, Xiangzhu Cun, Shiyu Lin, Taoran Tian, Bofeng Zhu, Yunfeng Lin

**Affiliations:** 1 State Key Laboratory of Oral Diseases, West China Hospital of Stomatology, Sichuan University, Chengdu, China; 2 Key Laboratory of Shaanxi Province for Craniofacial Precision Medicine Research, College of Stomatology, Xi'an Jiaotong University, Xi’an 710004, China; 3 Clinical Research Center of Shaanxi Province for Dental and Maxillofacial Diseases, College of Stomatology, Xi'an Jiaotong University, Xi’an 710004, China; 4 Department of Forensic Genetics, School of Forensic Medicine, Southern Medical University, Guangzhou 510515, China

## Abstract

Adipose-derived stromal cells (ASCs) have gained great attention in regenerative medicine. Progress in our understanding of adult neovascularization further suggests the potential of ASCs in promoting vascular regeneration, although the specific cues that stimulate their angiogenic behavior remain controversial. In this study, we established a three-dimensional (3D) angiogenesis model by co-culturing ASCs and endothelial cells (ECs) in collagen gel and found that ASC-EC-instructed angiogenesis was regulated by the canonical Wnt pathway. Furthermore, the angiogenesis that occurred in implants collected after injections of our collagen gel-based 3D angiogenesis model into nude mice was confirmed to be functional and also regulated by the canonical Wnt pathway. Wnt regulation of angiogenesis involving changes in vessel length, vessel density, vessel sprout, and connection numbers occurred in our system. Wnt signaling was then shown to regulate ASC-mediated paracrine signaling during angiogenesis through the nuclear translocation of β-catenin after its cytoplasmic accumulation in both ASCs and ECs. This translocation enhanced the expression of nuclear co-factor Lef-1 and cyclin D1 and activated the angiogenic transcription of vascular endothelial growth factor A (VEGFA), basic fibroblast growth factor (bFGF), and insulin-like growth factor 1 (IGF-1). The angiogenesis process in the 3D collagen model appeared to follow canonical Wnt signaling, and this model can help us understand the importance of the canonical Wnt pathway in the use of ASCs in vascular regeneration.

## Introduction

Adipose-derived stromal cells (ASCs) are believed to have great potential in vascular regeneration.^1^ The advantages of ASCs include a less invasive harvesting procedure, a larger number of stem cell progenitors from an equivalent amount of harvested tissue, better angiogenic, osteogenic, and adipogenic properties, and better immunomodulatory properties, which make ASCs a promising alternative to multi-functional bone mesenchymal stem cells.^2^ Recent studies in vascular regeneration has shown that ASCs promote endothelial tubulogenesis, contribute to neovascularization, and impact angiogenesis by secreting angiogenic cytokines and growth factors in a paracrine manner.^3^ ASCs showed a promising angiogenic effect in transplantations *in vivo* and in early clinical trials.^4,5^ However, the molecular mechanisms underlying these biological events are still heavily debated.

Multiple cell types involved in the regulation of angiogenesis express Wnt ligands.^6^ Animal models utlizing transgenic mice^7,8^ and tumor angiogenesis^9^ also implicate autocrine Wnt signaling as a vital regulator of angiogenesis. In addition to expressing Wnt ligands, vascular cells also respond to Wnt signaling via the canonical pathway.^10^ Recently, the canonical Wnt pathway has been implicated in many aspects of angiogenesis, vascular remodeling, and differentiation; it also influences vascular sprouting, network maturation, and arterio-venous specification through cell–cell interactions to consequently determine different cell fates.^8^ Although β-catenin-dependent Wnt signaling has been shown to regulate angiogenesis, the contribution of individual cell types in the activation of downstream cascade reactions in both endothelial cells (ECs) and themselves during blood vessel formation is still elusive.^7^

Lithium chloride (LiCl) is a canonical activator of Wnt signaling. Activation of the canonical Wnt pathway inhibits glycogen synthase kinase-3β from phosphorylating β-catenin, resulting in the accumulation of active β-catenin polypeptide species that translocate into the nucleus to activate transcription factors, such as Tcf/Lef.^11^ However, dickkopf-1 (DKK1) is a classic Wnt antagonist that binds to the Wnt receptors low-density lipoprotein receptor-related proteins 5 and 6 (LRP5/6), which thereby inhibits the formation of a ternary receptor complex to block β-catenin signaling.^12^ In addition to binding to LRP5/6, DKK1 also binds to Kremen (Krm)-1/2 with high affinity to inhibit Wnt/β-catenin signaling.^13^

Therefore, in the present study, we first established a three-dimensional (3D) collagen gel model and co-cultured ASCs and ECs to investigate whether angiogenesis was regulated by the canonical Wnt pathway. We applied LiCl and DKK1 in the co-culture system to further explore the influence of the Wnt pathway on the cellular mechanism of angiogenesis in both targeted ECs and paracrine ASCs. Identifying the contribution of individual cell types helps us understand the influence of the canonical Wnt pathway on ASCs in vascular regeneration.

## Materials and methods

Animal materials used for this study were obtained according to governing ethical principles, and approval from the Institutional Review Board (IRB) at the West China Hospital of Stomatology was obtained prior to commencing this study.

### Transgenic mice and cell culture

Transgenic mice (C57BL/6-Tg(CAG-EGFP)1Osb/J) that use the chicken β-actin promoter to direct the expression of enhanced green fluorescent protein (eGFP) were provided by the Center of Genetically Engineered Mice, West China Hospital, Sichuan University, Chengdu, China. Six mice were used for ASC isolation. The cell culture protocol for ASCs has been previously described.^14,15^ Briefly, the subcutaneous adipose tissue from 4-week-old female mice was collected aseptically, cut into small pieces and treated with 0.75% type I collagenase for 30 min. This ASC-containing suspension was then collected and mixed 1:1 (v/v) with fresh 10% heat-activated fetal bovine serum (FBS, Schaumburg, IL, USA), α-MEM (0.1 mmol·L^−1^ non-essential amino acids), 4 mmol·L^−1^ L-glutamine (HyClone, UT, USA), and 1% penicillin–streptomycin antibiotics. The mixed suspension was centrifuged at 180*g* for 5 min. After removing the upper supernatant containing adipose debris, 10% FBS α-MEM was added to re-suspend the ASCs. The suspension was added to plates or flasks at 37 °C in a humidified atmosphere of 5% CO_2_ for ASC attachment. Purified ASCs could then be obtained after two passages as previously described.^15^

Transgenic mice [Tg(CAG-DsRed MST)1Nagy/J] that express DsRed Express-positive protein (red fluorescent protein, RFP) were provided by the Genetic Centre of Institute of Laboratory Animal Sciences, Chinese Academy of Medical Sciences, and the Centre of Comparative Medicine, Peking Union Medical College, Beijing, China. Six mice were used for EC isolation. The cell culture protocol for ECs is described in a previous report.^4^ First, microvascular tissue was collected from neonatal mice. Tissue samples were cut into small pieces and treated with 0.5% type II collagenase for 1 h. The EC-containing suspension was collected and mixed 1:1 (v/v) with fresh 10% heat-activated FBS DMEM (high-glucose DMEM, 0.1 mmol·L^−1^ non-essential amino acids, 4 mmol·L^−1^ L-glutamine, 1% penicillin–streptomycin antibiotics), and the mixed suspension was centrifuged at 179*g* for 8 min. After removing the supernatant, the remaining tissue fragments were mixed with 20% bovine serum albumin and centrifuged at 1000*g* for 20 min. Once more, the supernatant was removed and fresh 10% FBS DMEM was added to centrifuge tubes to re-suspend the ECs. The EC-containing suspension was then added into plates or flasks at 37 °C in a humidified atmosphere of 5% CO_2_ for EC attachment. Passage 0 and 1 cells were used in the study. Angiogenesis studies were all based on the GFP-ASC and RFP-EC co-culture model.^16,17^

### Human cell culture

Human adipose stromal cells (hASCs) were obtained from human subcutaneous adipose tissue that was collected from adult male donors (55 years old) with the approval from the IRB at the West China Hospital of Stomatology. The hASCs were incubated in α-MEM containing L-glutamine supplemented with 10% FBS and 1% penicillin/streptomycin antibiotics at 37 °C in a humidified atmosphere with 95% air and 5% CO_2_. Cells from passage 2–4 were used in the study.

Human umbilical vein endothelial cells (HUVECs) were purchased from the American Type Culture Collection (ATCC^®^ CRL-1730™, ATCC, Manassas, VA, USA) and cultured in 10% FBS high-glucose DMEM with 1% penicillin/streptomycin antibiotics. The HUVECs were maintained at 37 °C in a humidified atmosphere (95% air and 5% CO_2_) until usage. Molecular analyses were all based on the hASC–HUVEC co-culture model.

### Cell identification

For identification, ASCs were cultured in six-well plates (2×10^5^ cells per well) with induced adipogenic media and osteogenic media (Biowit, Shenzhen, China) to show the differentiation abilities toward lipogenesis and osteogenesis. Lipid accumulations in the cells were visualized by Oil Red O staining, and osteogenic bone nodules were detected by Alizarin Red staining (Sigma-Aldrich, St. Louis, MO, USA).

Factor VIII (Biorbyt Limited, Cambridge, Cambridgeshire, UK) immunofluorescence was performed for the identification of ECs. ECs were cultured in 24-well plates (2×10^3^ cells per well) for 24 h and then permeabilized with 0.1% Triton X-100 (AMRESCO LLC, Solon, OH, USA) for 15 min and blocked with 1% bovine serum albumin for 20 min at 37 °C after three washes with phosphate-buffered saline (PBS). Afterwards, the cells were incubated at 4 °C overnight with a rabbit polyclonal antibody against factor VIII, after which they were incubated with FITC-conjugated donkey anti-rabbit IgG (Invitrogen, Carlsbad, CA, USA) for 1 h at 37 °C. Images were then captured using an IX71 inverted microscope (Olympus, Tokyo, Japan) and analyzed with Image-Pro Plus Software 6.0 (Media Cybernetics, Rockville, MD, USA).

### Flow cytometry

Third-passage ASC suspensions were prepared in 10% FBS in PBS. One set of test tubes contained cells stained with fluorescence-marked antibodies to CD34, CD44, CD146, and Sca-1; the other set of tubes without fluorescent antibodies was used as controls. We used anti-mouse CD34 and CD146 (both purchased from BioLegend, San Diego, CA, USA), fluorescence-labeled FITC-conjugated Sca-1 antibody (Abcam, San Francisco, CA, USA), and Alexa Fluor^®^ 488 anti-mouse CD44 (BioLegend). Tubes with CD44 and Sca-1 were incubated in the dark at room temperature (RT) for 30 min; simultaneously, tubes with CD34 and CD146 were incubated at RT for 1.5 h. Cells with directly labeled antibodies, including CD44 and Sca-1, were then washed in PBS (containing 10% FBS) and retained with other samples at 4 °C, whereas tubes with CD34 and CD146 were incubated in the dark with secondary antibodies (FITC goat anti-mouse IgG, Zhongshanjinqiao, Beijing, China) at RT for 30 min; then, the cells were washed again. Finally, PBS containing 10% FBS was separately added into all tubes, and cells were mixed with PBS. Analyses were performed using a fluorescence-activated cell sorter (FACSCalibur, BD Biosciences, San Diego, CA, USA), and data analysis was performed using WinMDI2.8 software (The Scripps Institute, La Jolla, CA, USA).

### Establishment of the 3D co-culture model

The GFP-ASCs from fat tissue and RFP-ECs from brain microvascular tissue were co-cultured in a 3D collagen gel model. The ASCs and ECs were co-cultured at a 1:1 ratio and suspended in high-glucose DMEM and rat tail tendon collagen type I (Shengyou Biotechnology, Hangzhou, China). The cell suspension was then transferred to the wells of a 96-well plate to form gels at 37 °C and cultured for 2 weeks. The images were captured with an IX71 inverted microscope (Olympus), and the vessel-like structures were quantified using Image-Pro Plus Software 6.0 (Media Cybernetics).

### Animal model

The 3D collagen gels with co-cultured ASCs and ECs were implanted into subcutaneous pockets at both dorsal sides of nude mice under ether inhalation anesthesia after being established and equilibrated in a 96-well plate for 12 h. The animals were divided into six groups, with four animals in each group. Treatments began on day 1 and continued for 2-week periods. Gel samples in each group (LiCl, DKK1, and PBS), which were the same as those used in the *in vitro* tests, were implanted at the backs of the nude mice. The implants were injected once per day with 0.1 mL of collagen containing DKK1, LiCl, or PBS.^14^ The collagen was then collected at 1 and 2 weeks for analysis.

### DKK1 and LiCl treatment

The effects of LiCl and DKK1 on *in vitro* angiogenesis in the 3D collagen gel were determined by co-culturing GFP-ASCs and RFP-ECs, and the expression of angiogenic factors in a transwell model were determined by co-culturing hASCs and HUVECs; DMEM medium containing either 11.794 mmol·L^−1^ LiCl (0.5 mg·mL^−1^; Sigma-Aldrich) or DKK1 (100 ng·mL^−1^; R&D Systems, Minneapolis, MN, USA) was used.

The effects of LiCl and DKK1 on *in vivo* angiogenesis in an implanted gel were determined using concentrations of 47.176 mmol·L^−1^ (2 mg·mL^−1^) per animal and 200 ng·mL^−1^ per animal, respectively.

### Immunofluorescence

The hASCs and HUVECs were cultured for 24 h in high-glucose DMEM containing either 11.794 mmol·L^−1^ of LiCl, 100 ng·mL^−1^ of DKK1, or PBS. The staining process was carried out as mentioned above using factor III stain. The cells were lastly counterstained with 4′6-diamidino-2-phenylindole, and images were captured using an IX71 inverted microscope (Olympus) and a modified confocal laser scanning microscope (TCS SP5, Leica, Wetzlar, Germany) and analyzed with Image-Pro Plus Software 6.0 (Media Cybernetics).

Cell images in 3D collagen were scanned using a modified Leica DMIRE2 confocal laser scanning microscope (Leica) equipped with a ×60 oil immersion objective lens. Imaris 7.0.0 (Bitplane, Zurich, Switzerland) was used for 3D reconstruction as previously described.^17^

### Histological and immunohistochemical staining

The implants from the backs of nude mice were fixed with 4% paraformaldehyde for at least 24 h and then embedded in paraffin and cut into 10-μm-thick sections at −20 °C. Hematoxylin and eosin (H&E) staining was performed to detect erythrocytes. For histological analyses, RFP and CD34 on the ECs from the capillaries in the tissue sections were stained using anti-RFP antibodies (ab62341 and ab81289, Abcam). The sections were then captured under the ECLIPSE 80i microscope (Nikon, Tokyo, Japan) and visualized using the NIS-Element F 3.0 Software (Nikon).

### Western blot

Protein samples were prepared by mixing one part of sample with one part of Bio-Rad Laemmli Sample Buffer and then boiled at 100 °C for 5 min. Proteins were separated in 8%–12% sodium dodecyl sulfate polyacrylamide gel electrophoresis (according to the molecular weights of the LOX profile) and transferred to a polyvinylidene difluoride membrane at 200 mA for 1 h at RT. The blot was blocked with 5% fat-free dry milk suspended in 1× TBST for 2 h at RT. The resulting blot was incubated with antibodies (1:500–1 000), including β-catenin/phosphorylated β-catenin, GAPDH, VEGFA, Lef-1, and cyclin D (Abcam), for 1–3 h at RT, followed by incubation with 1:5 000 anti-IgG-HRP (Alex Series, Abcam) for 2 h at RT. Signals from the blots were obtained using a Western Blotting Luminol Reagent Kit. Proteins were visualized via chemiluminescence with hydrogen peroxide using Kodak X-AR and luminol as substrates.

### Semi-quantitative polymerase chain reaction

RNA samples were isolated using the RNeasy Plus Mini Kit (Qiagen, Shanghai, China) with a genomic DNA eliminator. Isolated RNA was dissolved in RNase-free water and quantified by measuring the absorbance at 260 nm with a spectrophotometer. The RNA samples were then treated with DNase I (Mbi, Glen Burnie, MD, USA), and cDNA was prepared for each sample using 0.5 mg of total RNA and a cDNA synthesis kit (Mbi) in a final volume of 20 μL.

To evaluate the gene expression of VEGFs and growth factors normalized to the glyceraldehyde-3-phosphate dehydrogenase (GAPDH) gene, semi-quantitative PCR was performed with a PCR kit (Mbi) using a thermocycler (Bio-Rad, Hercules, CA, USA). BLAST was used to search for all primer sequences to ensure gene specificity. Semi-quantitative PCR reactions were performed in a 25-μL volume containing 1 μL of cDNA sample. PCR consisted of an initial 30-s denaturation step at 94 °C, 30 s of annealing at 55–65 and 72 °C, and 30 s of elongation with 20–28 amplification cycles. Products were resolved using 2% agarose gel electrophoresis in Tris-borate/ethylenediaminetetraacetic acid buffer and visualized by staining with ethidium bromide.

### Quantitative real-time PCR

Quantitative real-time PCR was performed with a Quanti-Tect SYBR Green PCR Kit (Qiagen) using an iCycler (Bio-Rad). PCR reactions were performed using 0.5 μmol·L^−1^ concentrations of each primer in a 25-μL volume containing 1 μL of cDNA sample. The reaction was initiated by activation with DNA polymerase with a 5-min pre-incubation at 95 °C. Amplification was achieved with 45 cycles of 15-s denaturation at 94 °C, 20 s of annealing at 65 °C, and 10 s of extension at 72 °C. The program was concluded by melting curve analysis. All experiments were performed in triplicate. The copy numbers of each gene were determined by cycle threshold (ΔCT) methods. The copy numbers of GAPDH were used as internal controls. Standard curves of all primers were prepared from total normal cDNA, which was amplified by semi-quantitative PCR and cloned using a TOPO II TA Cloning Kit (Invitrogen) following the manufacturer’s recommendations.

### Statistical analysis

Statistical data analysis was performed with SPSS 16.0 software using one-way analysis of variance to compare the mean values of all groups. The Student–Newman–Keuls test was used to compare the mean values of two groups. The data were considered statistically significant when the two-tailed *P*-values were less than 0.05.

## Results

### Angiogenesis by co-culturing ASCs and ECs in a 3D collagen model

**Figure 1:**
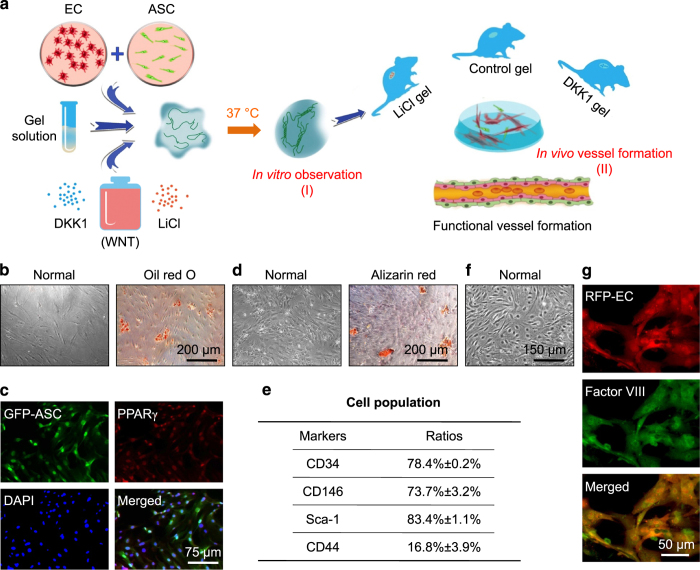
Experimental protocol and cell identification. (**a**) Schematic illustration showing total experimental protocol to establish a 3D vascular collagen model *in vitro* and *in vivo*. Briefly, GFP-ASCs and RFP-ECs were co-cultured at a 1:1 ratio and suspended in collagen matrices with a Wnt regulator, LiCl, or DKK1, and then gelled at 37 °C and studied *in vitro*. The gels were implanted into subcutaneous pockets at both dorsal sides of nude mice to set up an *in vivo* animal model for testing angiogenesis induced by Wnt regulators. Gels were collected and immediately macroscopically imaged by modified CLSM and then sectioned for histological and immunohistochemical analyses. (**b**–**e**) Identification of ASCs from mouse fat tissue. Oil Red O (**b**) and PPARγ (**c**) show adipogenic differentiation of isolated ASCs, and Alizarin Red (**d**) shows osteogenic differentiation. The stain images shown are representative of four different experiments (*n*=4). Flow cytometry (**e**) showing positive staining for CD34, CD146, and Sca-1 in isolated ASCs (*n*=3). (**f** and **g**) Identification of ECs from mouse brain microvascular tissue. Representative image (**f**) showing isolated primary ECs, and factor VIII immunofluorescence (**g**) showing EC marker staining in isolated ECs (*n*=3).

**Figure 2:**
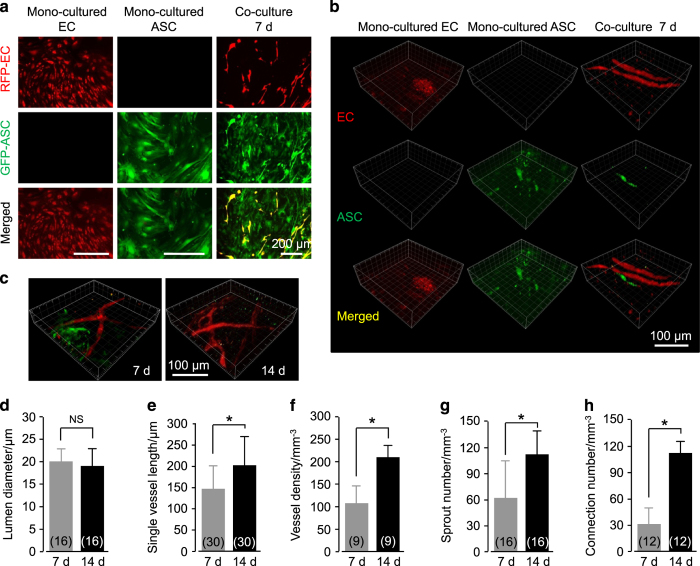
Angiogenesis in a 3D collagen model by co-culturing ASCs and ECs *in vitro*. (**a**) Formation of vessel-like structures in ASC-EC co-cultured 3D gels imaged by enhanced inverted microscopy. ASCs are GFP-positive and ECs are RFP-positive. The images shown are representative of four different experiments (*n*=4). (**b** and **c**) Formation of vessel-like structures in ASC-EC co-cultured 3D gels further imaged and reconstructed by modified CLSM. The images shown are representative of four different experiments (*n*=4). (**d**–**h**) Analyses of angiogenesis in ASC-EC co-cultured 3D gels. The numbers shown in parentheses indicate images (vascular-like structure diameter (**d**) and single vessel length (**e**)) examined in each case, and similar results were observed in four independent experiments (*n*=4). Analyses of vessel density (**f**), sprout number (**g**), and connection number (**h**) were performed for at least three independent experiments (*n*=3). *P<0.05; NS, no significant difference.

We first established an angiogenesis model *in vitro* by co-culturing ASCs from the fat tissue of enhanced GFP transgenic mice and ECs from the brain microvascular tissue of DsRed Express transgenic mice in a 3D collagen gel (Figures 1 and 2). We determined the potential for adipogenic differentiation by first examining the isolated ASCs by Oil Red O through 7 days of conditional media induction (Figure 1b) and by fluorescently staining the isolated GFP-ASCs with anti-PPARγ antibodies (Figure 1c). The potential for osteogenic differentiation was determined by examining the isolated ASCs by Alizarin Red through 7 days of conditional media induction (Figure 1d). The ASC population in isolated cells was determined by performing flow cytometry to confirm positive stromal cell markers, that is, CD34, CD146, and Sca-1 (Figure 1e). Primary ECs (Figure 1f) were used in the experiments for angiogenesis, and factor VIII immunofluorescence confirmed positive endothelial cell markers in isolated ECs (Figure 1g). We then mixed GFP-ASCs and RPF-ECs at a 1:1 ratio in a collagen gel and cultured the gel *in vitro* in a 96-well plate for 14 days. Particularly at 7 days, the co-cultured group formed vessel-like structures observed by enhanced inverted microscope, but the single ASC or EC group did not (Figure 2a). Three-dimensional images of the collagen gels were reconstructed from 20-layer scanning images by Imaris 7.0.0 to better show the vessel-like structure formation (Figure 2b). We found that more vessel-like structures formed at 14 days in comparison to angiogenesis at 7 days after co-culturing ASCs and ECs (Figure 2c). The diameter of vessel-like structures showed no significant differences at 14 days compared to those at 7 days (Figure 2d); however, after 14 days, the single vessel length (Figure 2e), vessel density (Figure 2f), sprout number (Figure 2g), and connection number (Figure 2h) were all increased to 155%, 192%, 180%, and 360%, respectively.

### ASC-paracrine angiogenesis is regulated by the canonical Wnt pathway

**Figure 3:**
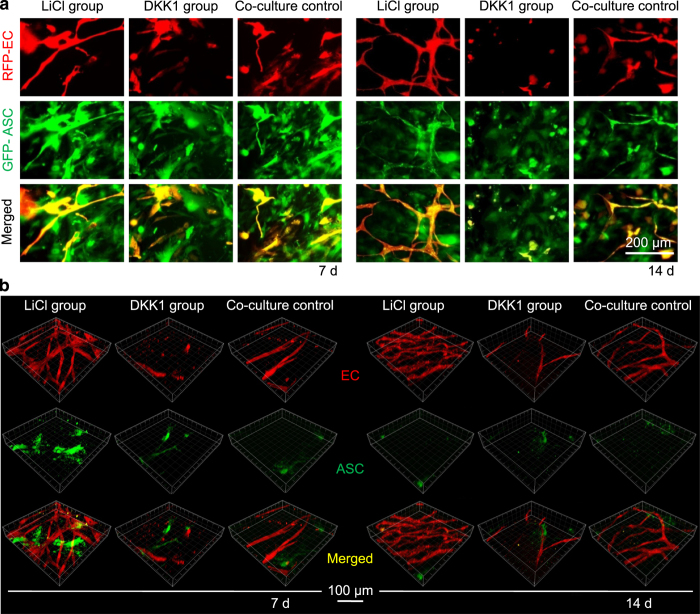
ASC-paracrine angiogenesis is modulated by Wnt regulators *in vitro*. (**a**) Formation of vessel-like structures in ASC-EC co-cultured 3D gels treated with DKK1 and LiCl by enhanced inverted microscopy. The left three groups show 7 days of treatments and the right three groups show 14 days of treatments. ASCs are GFP-positive and ECs are RFP-positive. The images shown are representative of four different experiments (*n*=4). (**b**) Formation of vessel-like structures in ASC-EC co-cultured 3D gels treated with DKK1 and LiCl imaged and reconstructed by modified CLSM. The left three groups show 7 days of treatments and the right three groups show 14 days of treatments. ASCs are GFP-positive and ECs are RFP-positive. The images shown are representative of four different experiments (*n*=4).

**Figure 4:**
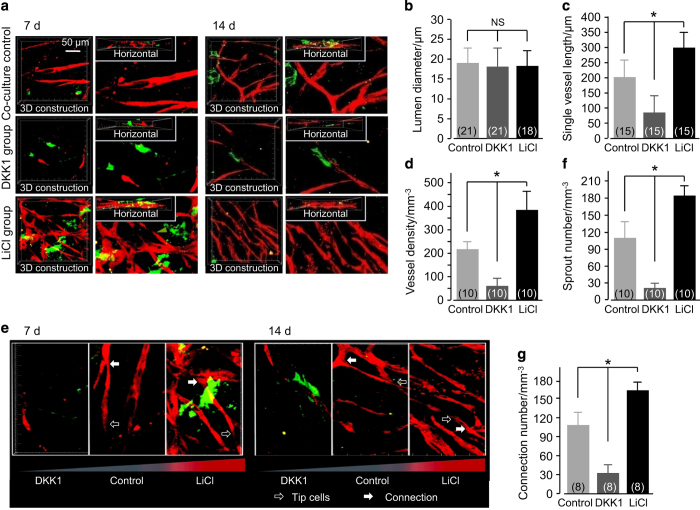
Single vessel lengths, vessel density, sprout number, and connection number in ASC-paracrine angiogenesis are regulated by Wnt regulators *in vitro*. (**a**) 3D reconstruction showing the morphologies of vessel-like structures regulated by DKK1 and LiCl in ASC-EC co-cultured 3D collagen gels. The regulation of vessel-like structures by DKK1 and LiCl at 7 days (left) and 14 days (right) are shown to be representative of four different experiments (*n*=4). (**b**–**d**) The parameter changes in ASC-paracrine angiogenesis by DKK1 and LiCl. The numbers shown in parentheses indicate images (vascular-like structure diameter (**b**) and single vessel length (**c**)) examined in each case, and similar results were observed for four independent experiments (*n*=4). The analysis of vessel density (**d**) was performed for least three independent experiments (*n*=3). *P<0.05; NS, no significant difference. (**e**) 3D reconstruction showing the sprouts and connections in vessel-like structures regulated by DKK1 and LiCl. The regulation of vessel-like structures by DKK1 and LiCl at 7 days (left) and 14 days (right) are shown to be representative of four different experiments (*n*=4). (**f** and **g**) Sprout number (**e**) and connection number (**f**) changes in ASC-paracrine angiogenesis by DKK1 and LiCl. The analyses of sprout number (**f**) and connection number (**g**) were performed for at least three independent experiments (*n*=3). *P<0.05; NS, no significant difference.

ASCs and ECs were co-cultured in 3D collagen gels with LiCl (11.794 mmol·L^−1^, 0.5 mg·mL^−1^), DKK1 (100 ng·mL^−1^), or PBS (the blank control group). After 7 and 14 days of *in vitro* incubation, the angiogenesis images were captured by enhanced inverted microscopy (Figure 3a). Compared with the blank co-culture group, LiCl increased the number of extended branches and vessel-like structures that protruded from the ECs at 7 days. However, DKK1 could not induce the formation of these structures, and most ECs remained as single cells (Figure 3a). The densities of vessel-like structures increased with LiCl treatment at 14 days, which caused the endothelial cells to extend their branches to connect with one another and form advanced vessel-like structural networks; however, as with the DKK1-treated group, cells did not form connections (Figure 3a). The collagen gels were taken out from the 96-well plate and the angiogenesis images were captured by modified confocal laser scanning microscope (CLSM) to obtain further convincing evidence of angiogenesis. We found clear angiogenesis modulated by LiCl and DKK1 by 3D reconstruction from 20-layer scanning CLSM images (Figure 3b). Analysis of the reconstruction images (Figure 4a) showed no significant differences in the diameters of vascular-like structures formed among the control, LiCl, and DKK1 (Figure 4b); however, the single vessel length (Figure 4c) and vessel density (Figure 4d) significantly increased in the LiCl group (to 150% and 172%, respectively) and decreased in the DKK1 group (to 42% and 28%, respectively) compared to those in the control groups. We analyzed the sprouts and connections in the vessel-like structures (Figure 4e) and found that more sprouts (Figure 4f) and connections (Figure 4g) emerged in the LiCl group (increases to 168% and 155%, respectively) than in the control groups, but there were relatively fewer in the DKK1 group (reductions to 18% and 28%, respectively).

### ASC-paracrine functional angiogenesis is further regulated by the canonical Wnt pathway

**Figure 5:**
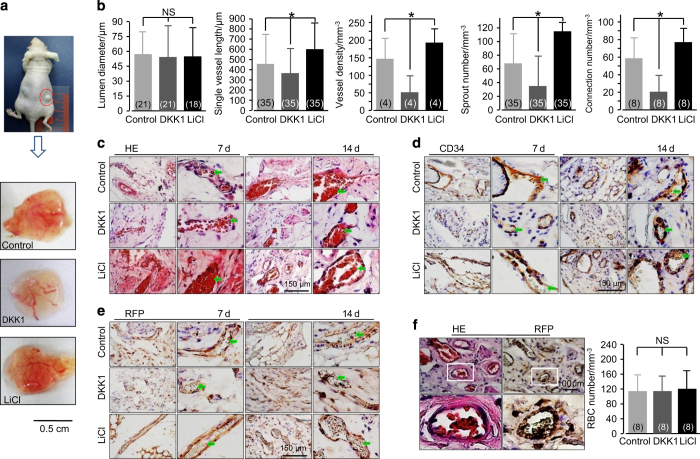
The *in vivo* capillary development of the implants is modulated by the Wnt pathway. (**a**) Representative morphologies of capillaries in the implanted gels (14 days) collected from the subcutaneous pockets at both dorsal sides of nude mice are regulated by DKK1 and LiCl. (**b**) Analyses of angiogenesis in the implants. The numbers shown in parentheses indicate images (vascular-like structure diameter and single vessel length) examined in each case and similar results were observed for four independent experiments (*n*=4). The analyses of vessel density, sprout number, and connection number were performed for at least three independent experiments (*n*=3). *P<0.05; NS, no significant difference. (**c**) H&E stain showing newly formed capillaries with red blood cells inside. The images shown are representative of four experiments (*n*=4). (**d**) Positive immunohistochemical staining of RFP implies that these newly formed functional capillaries in the sections originated from the implanted endothelial cells. The images shown are representative of four experiments (*n*=4). (**e**) Positive immunohistochemical stain of CD34 indicates that these newly formed functional capillaries in the sections originated from the implanted cells. The images shown are representative of four experiments (*n*=4). (**f**) H&E stain and RFP stain from one implanted gel section indicates that these newly formed functional capillaries originated from the implanted endothelial cells. The images shown are representative of four experiments (*n*=4).

After co-culturing ASCs and ECs in 3D collagen gels with LiCl (47.176 mmol·L^−1^, 2 mg·mL^−1^), DKK1 (200 ng·mL^−1^), or PBS (the blank control group) 12-h post equilibration, the gel samples were implanted into subcutaneous pockets at both dorsal sides of nude mice. At 7 and 14 days, the nude mice were killed, and implants were collected. The different morphologies of angiogenesis regulated by LiCl and DKK1 are shown (Figure 5a). Lumen diameters among these three groups showed no significant differences (Figure 5b); however, the single vessel length and vessel density significantly increased in the LiCl group (to 126% and 128%, respectively) and decreased in the DKK1 group (to 43.5% and 35.6%, respectively) compared with those in the control groups. Additionally, the vascular structures extended many sprouts and connections with other branches in the LiCl group (Figure 5b). New sprouts and branches were also observed in the control group, but fewer branch connections were noted. Only a few vessel branches were observed in the DKK1 group.

The collected implants were then frozen and sectioned, and the sample slices were immunohistochemically stained. From H&E staining at 7 and 14 days, we found that red blood cells were observed in these newly formed capillaries in all groups (Figure 5c), meaning that this ASC-paracrine angiogenesis was functional. The immunohistochemical staining using RFP then showed that these functional vessel structures originated from the ECs that were implanted *in vivo* (Figure 5d). We used another marker, CD34, and further confirmed that these vessel structures originated from the implanted cells (Figure 5e). Although LiCl and DKK1 could modulate the single vessel length, vessel density, sprout number, and connection number, there were no significant differences in the number of red blood cells per cubic millimeter (Figure 5f).

### Wnt regulates ASC-paracrine functional angiogenesis via nuclear translocation of β-catenin

**Figure 6:**
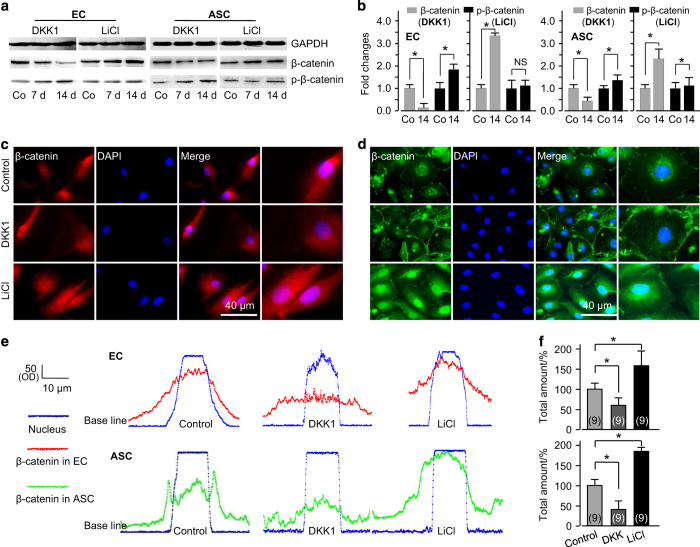
Wnt-regulated ASC-paracrine angiogenesis is based on nuclear translocation of β-catenin. (**a**) Western blot showing that β-catenin/phosphorylated β-catenin was regulated by DKK1 and LiCl in both ECs and ASCs. The gels shown are representative of three different experiments (*n*=3). (**b**) Quantified analyses of β-catenin/phosphorylated β-catenin regulated by DKK1 and LiCl in ECs and ASCs at 14 days using Quantity One 4.6.3 software. *P<0.05; NS, no significant difference. (**c**) Immunofluorescent stain showing the nuclear accumulations of β-catenin in ECs and ASCs at 14 days after treatment with LiCl or DKK1. The images shown are representative of three different experiments (*n*=3). (**d**) Representative linear fluorescence quantification showing the accumulation of nuclear translocated β-catenin in ECs and ASCs regulated by DKK1 and LiCl. (**e** and **f**) Total amounts of β-catenin translocated into the nucleus in both ECs and ASCs. The data shown are representative of nine different experiments (*n*=9). *Significant difference with respect to the control group (*P*<0.05).

We elucidated the mechanism of how LiCl and DKK1 modulate ASC-paracrine functional angiogenesis by focusing on β-catenin using transwell co-culture between HUVECs and human ASCs (hASCs). After 7 and 14 days of co-culture, we first detected the total protein expression of β-catenin and phosphorylated β-catenin in HUVECs and hASCs (Figure 6a). We found that LiCl significantly increased total β-catenin accumulation. However, DKK1 decreased β-catenin accumulation and promoted phosphorylated β-catenin (Figure 6b). We further confirmed by immunofluorescence that LiCl promoted the nuclear translocation of β-catenin in both HUVECs and hASCs, but DKK1 eliminated this phenomenon (Figure 6c (HUVECs) and Figure 6d (hASCs)). We found higher nuclear accumulation of β-catenin in the LiCl group and lower accumulation in the DKK1 group in both HUVECs and hASCs through fluorescence quantification using ImageJ software (Figure 6e). The total amounts of β-catenin translocated into the nucleus increased by up to 65% in HUVECs and 78% in hASCs in the LiCl group, but they decreased by up to 42% in HUVECs and 60% in hASCs (Figure 6f).

### β-catenin nuclear translocation activates a profile of growth factors related to angiogenesis

**Figure 7:**
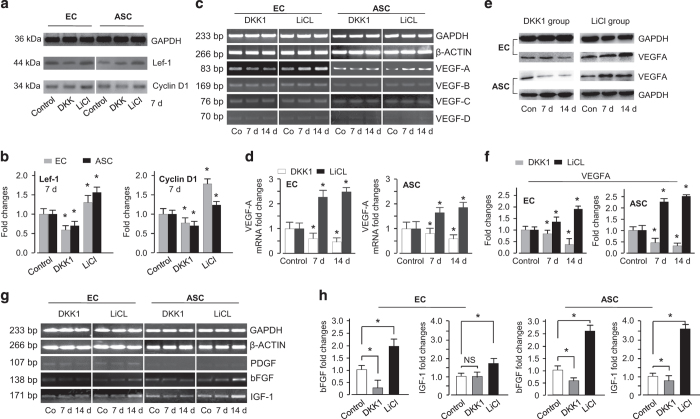
Nuclear translocation of β-catenin regulates Lef-1 and cyclin D and activates the transcriptional targets of growth factors. (**a** and **b**) The expression of Lef-1 and cyclin D regulated by DKK1 and LiCl in ECs and ASCs as shown by western blotting (**a**) and OD quantification with Quantity One 4.6.3 software (**b**). The data shown are representative of three different experiments (*n*=3). *P<0.05. (**c**–**f**) Nuclear translocation of β-catenin activates VEGFA expression. (**c**) Semi-quantitative PCR screened for the gene changes of VEGFA in ECs and ASCs regulated by DKK1 and LiCl. The gels shown are representative of three different experiments (*n*=3). Quantitative real-time PCR (**d**) confirmed VEGFA transcriptional changes in both ECs and ASCs. The images are representative of three different experiments (*n*=3). *P<0.05. Western blot (**e**) shows the protein expression of VEGFA in ECs and ASCs regulated by DKK1 and LiCl. OD quantification (**f**) confirmed protein changes (*n*=3). *P<0.05. (**g** and **h**) Nuclear translocation of β-catenin activates gene changes of growth factors, that is, PDGF, bFGF, and IGF-1, by semi-quantitative PCR (**g**) and quantitative real-time PCR (**h**). The gels (**g**) and experiments (**h**) shown are representative of three different experiments (*n*=3). *P<0.05.

Next, we found that β-catenin nuclear translocation first activated Lef-1 and cyclin D1 (Figure 7a). In the LiCl group, Lef-1 and cyclin D1 increased to 129% and 172% in HUVECs and 133% and 125% in hASCs, respectively; however, in the DKK1 group, both Lef-1 and cyclin D1 decreased (down to 63% and 79% in HUVECs and 75% and 59% in hASCs, respectively) (Figure 7b). We further examined the VEGF gene expression profile regulated by this cascade reaction caused by β-catenin nuclear translocation. We found that the VEGFA gene increased in the LiCl group and decreased in the DKK1 group, but VEGFB, C, and D did not significantly change (Figure 7c). Quantitative real-time PCR confirmed that the VEGFA gene increased to 247% in HUVECs and 181% in hASCs at 14 days (Figure 7d). We then detected the protein expression of VEGFA (Figure 7e), and the results were consistent with those for the VEGF genes (increased to 176% and 255% in the LiCl group, decreased to 38% and 25% in the DKK1 group in HUVECs and hASCs at 14 days, respectively) (Figure 7f). Additionally, we screened the gene profile of growth factors and found that bFGF and IGF-1 were both upregulated, as shown by both semi-quantitative PCR (Figure 7g) and quantitative real-time PCR (bFGF increased to 210% and 260% in the LiCl group and IGF-1 increased to 170% and 363% in the LiCl group by semi-quantitative PCR and quantitative real-time PCR, respectively) (Figure 7h).

## Discussion

Recent research progress suggests a potential role of ASCs in neovascularization.^18^ ASCs and bone mesenchymal stem cells help with vessel formation by co-culture with HUVECs or endothelial progenitor cells.^19^ Although this revascularization phenomenon is promising, the specific cues that stimulate the ASCs’ angiogenic behavior are poorly understood. In this study, we first confirmed that ASCs promote angiogenesis in the same way as co-culture with HUVECs or endothelial progenitor cells by co-culturing ASCs with ECs in a 3D collagen gel model. Then, we further revealed that canonical Wnt signaling through the nuclear translocation of β-catenin targeted both ECs and ASCs to regulate angiogenesis.

Angiogenesis is the sprouting of microvessels from a preexisting capillary network, and the process involves a harmonized interplay between various angiogenic and antiapoptotic factors.^20^ Herein, we co-cultured ASCs with ECs in a 3D collagen gel, which we found to be effective for angiogenesis. This formation of vessel-like structures is similar to previous reports of fibrin scaffolds with co-culture of ECs with human primary osteoblasts,^20^ ASCs with HUVECs, or endothelial progenitor cells with mesenchymal progenitor cells derived from bone marrow.^21^ Furthermore, we confirmed that the formation of vascular-like structures and new vessel sprouts increased and that new vessel networks gathered, indicating the likely formation of mature blood vessels undergoing angiogenic sprouting, vascular network stabilization, and hierarchical vessel perfusion. However, we had no data to support that ASCs, besides their function in paracrine secretion, became pericytes and actively participated in the formation of mature vessels, especially in the functional angiogenesis in implants *in vivo*.

LiCl, as an effective activator of Wnt/β-catenin signaling, is a competitive inhibitor of Mg^2+^ and can directly inhibit the Mg^2+^-ATP-dependent catalytic activity of glycogen synthase kinase-3β.^22,23^ The inhibition of glycogen synthase kinase-3β increases the cytosolic accumulation of β-catenin.^24^ This effect is consistent with our results regarding β-catenin localization. β-catenin accumulation in the cytoplasm promotes its translocation into the nucleus, where it binds the co-factor Tcf/LEF-1^25^ to stimulate gene transcription.^26,27^ However, the Wnt antagonist DKK1 binds to Wnt receptors LRP5/6 and thereby inhibits the formation of a ternary receptor complex, resulting in the blockade of β-catenin signaling.^28,29^ In the present study, the DKK1 group showed downregulated expression of total β-catenin, thus little was shown in nuclear translocation. This Wnt-modulated angiogenic process in our study followed the basal regulation principle of canonical Wnt signaling.^30^

The accumulation of active β-catenin results in its translocation into the nucleus and activates a group of angiogenesis-related genes.^31^ We first confirmed the increase of Lef-1 and cyclin D1 in ECs and ASCs after co-culture and detected the upregulation of VEGFα, a key growth factor acting through endothelial tyrosine kinase receptors, to promote the induction of the tip cell phenotype and the stimulation of both tip cell migration and stalk cell proliferation.^32^ After screening the growth factors, we also found that bFGF and IGF-1 significantly increased in both ECs and ASCs, which suggested that these growth factors might be important paracrine factors in ASC-aided functional angiogenesis.^33,34^

We acknowledge that there are two major limitations to this study that need to be further addressed in future investigations. First, ASCs in the co-culture system of ASCs and ECs could differentiate into endothelial cells to contribute angiogenesis, as previously reported.^35–38^ However, in this study, we observed vessel-like structure formation in ASCs and the nuclear translocation of β-catenin in ASCs; thus, further study is needed to confirm this result. Second, we could not clarify whether the functional capillaries formed *in vivo* were arterial or venous, even though Wnt/β-catenin signaling might modulate the Notch pathway to influence arterio-venous specification.^39–41^

Taken together, our data have shown that the angiogenesis process in the 3D collagen model of ASC and EC co-culture generally followed the regulation principles of canonical Wnt signaling and that this model is helpful in investigating the mechanisms of the canonical Wnt pathway in regulating ASC-mediated vascular regeneration.
